# Standardised images of novel objects created with generative adversarial networks

**DOI:** 10.1038/s41597-023-02483-7

**Published:** 2023-09-02

**Authors:** Patrick S. Cooper, Emily Colton, Stefan Bode, Trevor T.-J. Chong

**Affiliations:** 1 Turner Institute for Brain and Mental Health, Monash University, Victoria, 3800 Australia; 2 Melbourne School of Psychological Sciences, University of Melbourne, Victoria, 3010 Australia; 3 Department of Neurology, Alfred Health, Melbourne, Victoria, 3004 Australia; 4 Department of Clinical Neurosciences, St Vincent’s Hospital, Victoria, 3065 Australia

**Keywords:** Human behaviour, Psychology

## Abstract

An enduring question in cognitive science is how perceptually novel objects are processed. Addressing this issue has been limited by the absence of a standardised set of object-like stimuli that appear realistic, but cannot possibly have been previously encountered. To this end, we created a dataset, at the core of which are images of 400 perceptually novel objects. These stimuli were created using Generative Adversarial Networks that integrated features of everyday stimuli to produce a set of synthetic objects that appear entirely plausible, yet do not in fact exist. We curated an accompanying dataset of 400 familiar stimuli, which were matched in terms of size, contrast, luminance, and colourfulness. For each object, we quantified their key visual properties (edge density, entropy, symmetry, complexity, and spectral signatures). We also confirmed that adult observers (N = 390) perceive the novel objects to be less familiar, yet similarly engaging, relative to the familiar objects. This dataset serves as an open resource to facilitate future studies on visual perception.

## Background & Summary

Object perception has long been an area of interest in the visual neurosciences^1–10^. When viewing a scene, our attention is typically captured by objects that are salient (which may be driven by low-level visual features such as contrast^11–13^), and guided by our expectations (based on prior knowledge of the objects themselves^13,14^). A particularly topical question is how the brain processes object information in the absence of any such prior beliefs. Examining how perceptually novel objects are processed, and what features of those objects guide curiosity, exploration and learning^14–22^,, therefore has the potential to provide significant insights into the structure of the visual hierarchy.

Although several studies have focused on the processing of perceptually novel objects, reconciling their disparate findings has been challenging because of the heterogeneity in stimulus sets used. For example, some studies have used stimuli that are abstract (e.g., shapes, fractals) or configurally impossible^16,18–20,23–25^. A limitation of such stimuli is that they differ fundamentally in their structure or meaning relative to everyday objects, and may consequently be less engaging to an observer. Other studies have used: objects that are simply less common; everyday items photographed in unusual configurations; or different arrangements of familiar elements (e.g., blocks or plastic shapes)^25,26^. However, such approaches risk the diametrically opposite issue of all objects being potentially considered familiar and recognisable.

To understand how an object is visually processed independent of any prior expectations requires a set of objects that is: (1) realistic; (2) novel (i.e., could not possibly have been previously encountered); and (3) no less engaging than more familiar objects. Furthermore, in order to compare the visual processing of novel vs familiar objects, it is critical to ensure that differences in object type are not confounded by low-level visual features, such as size, contrast, luminance, or colourfulness. Although there are several well-curated datasets of familiar, real-world objects^27–32^, there is only one comparable dataset of novel objects (the Novel Object and Unusual Name Database; NOUN^26^). However, this dataset comprises only 64 objects, most of which are unusual (rather than truly novel) objects made of simpler shapes. Thus, there remains a conspicuous lack of standardised stimuli to facilitate research into object novelty.

To fill this gap, we trained Generative Adversarial Networks (GANs) – a class of machine learning algorithm – on everyday objects, with the goal of integrating their features to generate a set of 400 entirely novel, yet realistic, stimuli. To facilitate comparisons between the processing of novel and familiar objects, we curated an accompanying set of everyday objects sourced from existing open-source datasets (the Bank of Standardized Stimuli, BOSS^33,34^) and free online images (see Attributions in the dataset), and carefully matched their key low-level perceptual features (e.g., size, contrast, luminance, colourfulness). In an online study of 390 adult observers, we then confirmed that these novel stimuli were subjectively less familiar, but similarly engaging, relative to the familiar stimuli. The outcome is ‘IMAGINE’ (IMages of AI-Generated Imaginary Novel Entities) – a well-matched, standardised dataset of novel and familiar objects that can be readily applied in future investigations of object perception and perceptual novelty.

## Methods

### Stimulus generation

#### Novel objects

Novel objects were created using the *artbreeder* website (https://artbreeder.com), which generates artificial images of objects using a GAN algorithm. GANs are a class of machine learning algorithm where learning occurs via a competition in a *minmax* two-player game between two models: a *generator* model that attempts to produce a data sample that replicates the underlying latent properties of a training dataset, and a *discriminator* model that estimates the likelihood of a data sample being originally part of the training data^35^. That is, the generator’s goal is to maximise the number of false positives the discriminator produces, while the discriminator aims to increase its classification accuracy. Efficacy for these goals are represented within separate loss functions for the generator and discriminator, which are then used to improve subsequent training rounds. Ultimately, GANs produce data that is comparable to the original training data using this competition scenario. In the case of images, this results in a generator network that has learnt how to model a data distribution that can produce artificial images (e.g., objects) which appear as though they were part of the real, training set.

*Artbreeder* applies the commonly used *BigGAN*^36^ and *styleGAN*^23^ architectures to generate artificial images of objects. First, an image is created using these GANs, with the option to restrict the latent space sampled (truncation). Next, using Compositional Pattern Producing Networks (CPPNs)^37^, predefined networks that produce known classes of images (“genes”) can be added to the original “parent” image network to produce a new image that includes both features from the original and the selected “genes”. In practice, this allows one image (e.g., a dog) to be “bred” with other types of images (e.g., a toaster) to produce a hybrid dog*toaster object. Importantly, CPPNs allow the “genes” that produce the resultant image to be independently altered. Thus, different variations of an image can be created by bespoke tweaking of specific genes.

For this dataset, each novel object was based on a different set of parents and genes. We selected parent images from *artbreeder*’s “general” class that had white/light-coloured backgrounds, which were more likely to produce images of single objects, rather than scenes. We then trained the GANs on the selected parent images, and pseudo-randomly selected genes that maximised the likelihood of creating distinct novel objects. We generated an initial set of 761 novel images. As a quality control measure, each image was manually inspected and assessed by one or more of the authors (PC and/or EC) for:

1. Clarity – Stimuli were excluded if they were poorly defined (i.e., lacking clear form or internal detail);
2. Similarity to other objects – We ensured that the generated images were distinct from commonplace objects, or other novel or familiar objects in the dataset;
3. Artefacts and fragments – Occasionally, during the image creation process, a well-defined primary object would be accompanied by blurred or indistinct shadows or fragments separate to the object itself. We used image-editing software to remove any such artefacts from the final image (GNU Image Manipulation Program; GIMP).

**Fig. 1:**
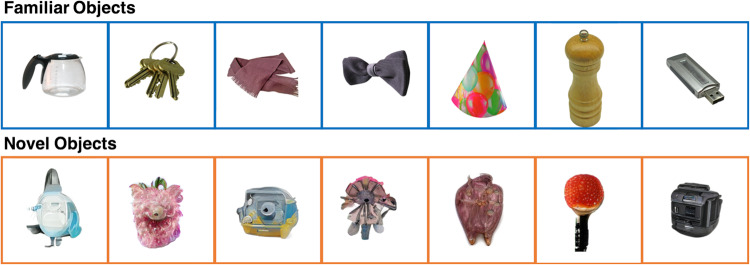
Exemplars of familiar and novel objects contained in IMAGINE.

This resulted in a curated data set of 400 novel objects (see Figs. 1, 5 for exemplar images).

#### Familiar objects

To benchmark the properties of these novel stimuli to those of real-life objects, we curated a companion data set comprising an equivalent number of familiar objects. We pooled images from the Bank of Standardised Stimuli (BOSS; both Phase I and II)^33,34^, as well as online sources of appropriately licenced images. The BOSS dataset comprises a set of standardised images, which were photographed and digitally edited to ensure a) only the object remained in frame and b) luminance and colouring were equalised^33,34^. The familiarity of the BOSS images has been previously confirmed by having participants rate their familiarity with the objects, and assessing the degree to which participants agree on the names of the objects.

For our purposes, we manually inspected all items in the BOSS dataset, and excluded those that were dated (e.g., slides, camera film), affectively valenced (e.g., weapons, religious iconography), or highly culturally specific (e.g., American football helmet). We also selected objects that were generally of a similar shape to our novel stimuli, which tended to be confined to the central portion of the canvas (e.g., we excluded elongated objects, such as broom, ruler). We supplemented the BOSS images with those from other internet sources, which were selected with similar criteria (attributions are provided in the released dataset).

#### Image standardisation

First, we standardised the image size (in pixels) of all novel and familiar objects. We used cubic interpolation implemented in GIMP to ensure that all objects were 150,000 pixels +/− 10% in size. We ran any image whose quality was reduced following resizing (35 novel objects) through the auto-enhancement procedure from Claid Studio (https://claid.ai). All object backgrounds were recoloured to white. Each object’s canvas was cropped to include a 100-pixel white border around its perimeter.

After standardising the size of each object, we ensured that the set of novel and familiar stimuli were closely matched in terms of key perceptual features. Specifically, we selected a subset of familiar objects that matched the novel objects in terms of:

1. Contrast – The standard deviation of the normalised, greyscale image (i.e., the standard deviation of the intensity values for the image);
2. Luminance – The average of the normalised greyscale image;
3. Colourfulness – The linear combination of the mean and standard deviation of the object’s colour plane^38^. To compute this value, we first constructed a simple opponent colour space for each image (comprising rg [red-green] and yb [yellow-blue]).
  $$rg=\left|R-B\right|$$
  $$yb=\frac{1}{2}\left(R+G\right)-B$$
  Next, we computed the mean and standard deviation of both *rg* and *yb* colour spaces:
  $${\mu }_{rgyb}=\sqrt{{\mu }_{rg}^{2}}+{\mu }_{yb}^{2}\,;{\sigma }_{rgyb}=\sqrt{{\sigma }_{rg}^{2}+{\sigma }_{yb}^{2}}$$
  We then defined colourfulness as:
  $$Colourfulness={\sigma }_{rgyb}+\left(0.3\times {\mu }_{rgyb}\right)$$
  In addition, we computed other image properties that are known to influence perceptual processing^38–43^. These include:
4. Edge density – The proportion of the total image space that consists of edges, as defined by the Canny^44^ procedure implemented with MATLAB’s *edge* function;
5. Entropy – The degree of randomness in an image, which provides a measure of the average amount of information it contains. Entropy, *H*, was defined in terms of the Shannon entropy function: $H=-\sum \left({P}_{k}\cdot lo{g}_{{P}_{k}}\right)$, where *P*_*k*_ is the probability of image pixels with value *k*. Images were converted to greyscale prior to calculation, so that *k* reflects image intensity, as implemented with *skimage’s* Shannon entropy function^45^;
6. Symmetry^39^ – The discrepancy in the number of pixels of two halves of an object split along multiple planes by applying a radon transformation on the image. Radon transformation was applied using *skimage’s* radon transformation, with symmetry computed as the mean of the transformation;
7. Object complexity – We applied two separate, commonly-used algorithms that define complexity as a function of the number of distinct features embedded within an object:
  7.1. Maximally Stable Extremal Regions (MSER) – This algorithm detects ‘distinct features’ as areas of an image with consistent intensity values. That is, consistent intensity values reflect patches of “texture” on an image, with more textures associated with a more complex image. We used MATLAB’s *detectMSERFeatures* algorithm.
  7.2. Scale-Invariant Feature Transform (SIFT)^46^ – This defines the number of ‘key points’ in an image based on features of an image that are robustly detected when the image is rescaled or re-orientated. We employed the OpenCV implementation of the SIFT algorithm.
8. Spectral energy – A common approach to control for low-level visual confounds in psychophysics and neuroimaging experiments is to ensure that stimuli are matched in spectral energy^38^. We used the Natural Image Statistical Toolbox for MATLAB^42^ with default parameters to measure the spectral profile of each image in terms of its:
  8.1 Image energy – The frequency that contains 80% of the image energy.
  8.2 High spatial frequencies – The proportion of high spatial frequencies in the image (>10 cycles per image).

## Data Records

**Fig. 2:**
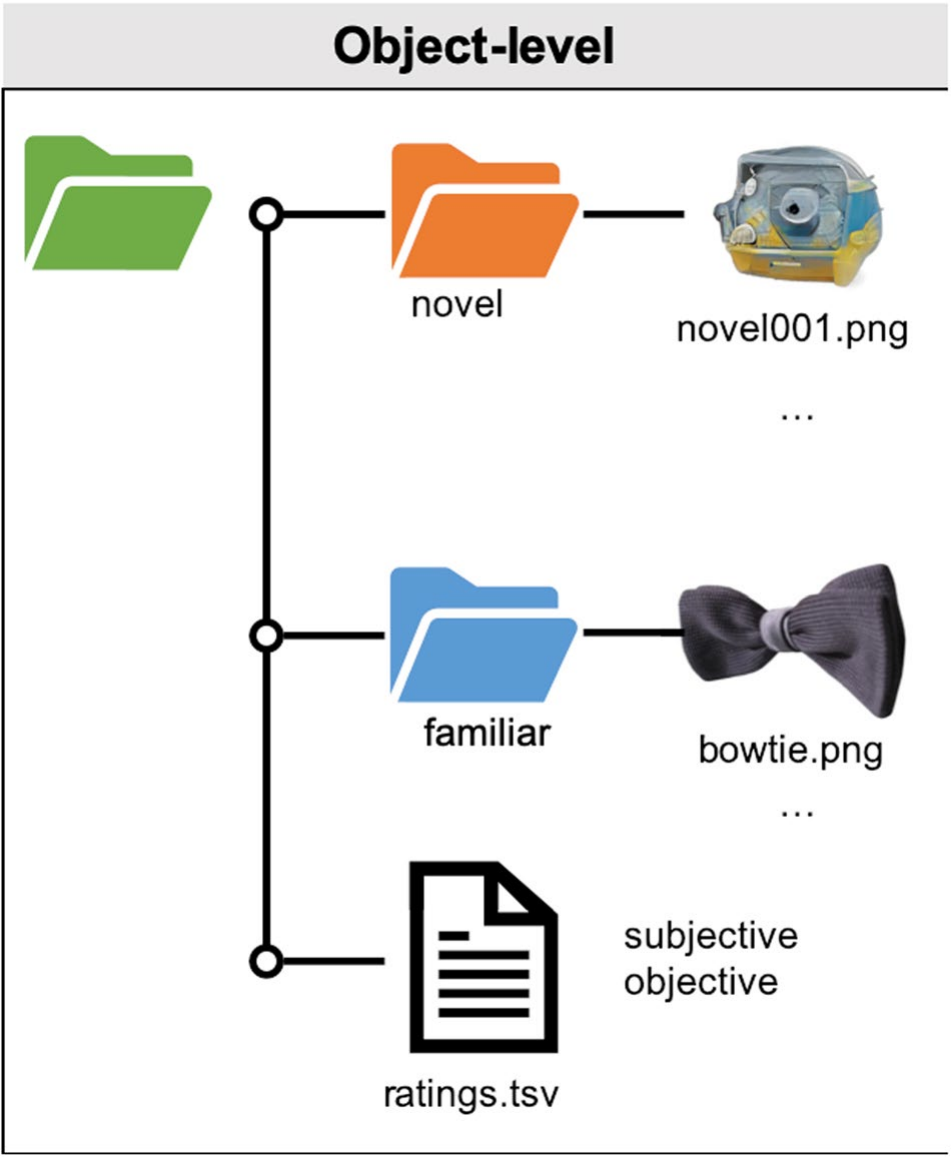
Dataset structure. Data are organised at the item-level, with separate folders for JPEG and PNG formats.

Data are stored using the Open Science framework (OSF) online platform as the IMAGINE dataset^47^. The released dataset contains standardised images of 400 novel and 400 familiar objects. Novel and familiar objects have been sorted into their respective folders (Fig. 2). Filenames for novel objects are prefixed by ‘novel’, followed by a three-digit code (e.g., ‘novel001’). Filenames for familiar objects comprise their original filenames from the BOSS dataset^33,34^ (e.g., *aceofdiamond*) or a descriptor if sourced from other repositories (e.g., *bananas*). All images are provided in compressed *JPEG* format (with a white background) and as *PNG* files (with a transparent background). An accompanying ratings.tsv file contains a summary of the perceptual properties of each object, and data from the online validation study that provides mean familiarity, interest and appeal ratings of every object.

## Technical Validation

Our technical validation comprised two parts. First, we confirmed that our corpuses of novel and familiar objects were closely matched in terms of size, contrast, luminance, and colourfulness. Second, we verified that our novel images were indeed perceived by human observers to be subjectively less familiar than the images of existing objects, and evoked comparable levels of interest.

### Standardisation of object properties

Many metrics violated assumptions of normality and so we compared perceptual features of the novel vs familiar objects using non-parametric statistics (Mann-Whitney U). We report both frequentist statistics, and their Bayesian analogues to guide interpretations of any differences between object categories (Figs. 4, 5). Critically, there were no differences between the novel and familiar objects in terms of their size, contrast, luminance, or colourfulness (size, novel_median_ = 146,464 ± 407 SEM, familiar_median_ = 146,020 ± 364, U = 79542, p = 0.889, BF_10_ = 0.081; contrast, novel_ = _0.27 ± 0.003, familiar_ = _0.27 ± 0.003, U = 79478, p = 0.873, BF_10_ = 0.078; luminance, novel_ = _0.83 ± 0.003, familiar_ = _0.84 ± 0.003, U = 77312, p = 0.411, BF_10_ = 0.100; colourfulness, novel_ = _11.3 ± 0.720, familiar = 9.9 ± 0.729, U = 75491, p = 0.168, BF_10_ = 0.183; Fig. 4a). This confirms that our approach was able to generate a set of novel objects that were matched in terms of their key lower level perceptual features to a set of existing familiar objects.

For completion, we also computed the more complex perceptual features of each object. We found that, relative to familiar objects, our set of novel objects had greater edge densities, greater entropy, less symmetry, greater complexity, and higher spectral energy (edge densities, novel_ = _0.056 ± 6.63 × 10^−4^, familiar_ = _0.052 ± 8.83 × 10^−4^, U = 66245, p < 0.0001, BF_10_ = 207; entropy, novel_ = _3.61 ± 0.021, familiar = 3.09 ± 0.029, U = 37686, p < 0.0001, BF_10_ = 1.22 × 10^11^; symmetry, novel = 91.9 ± 0.376, familiar = 94.2 ± 0.351, U = 69190, p < 0.0001, BF_10_ = 14.3; MSER, novel_ = _179 ± 10.7, familiar_ = _120 ± 20.4, U = 62511, p < 0.0001, BF_10_ = 462; SIFT keypoints, novel_ = _769 ± 34.4, familiar_ = _404 ± 36.1, U = 48764, p < 0.0001, BF_10_ = 1.56 × 10^7^; image energy, novel = 5 ± 0.27, familiar = 4 ± 0.36, U = 66059, p < 0.0001, BF_10_ = 38.4; high spatial frequencies, novel = 10.6 ± 0.42, familiar = 8.74 ± 0.49, U = 65810, BF_10_ = 75.3; Fig. 4a).

Finally, we ensured that the GAN-generated objects were indeed perceived as less familiar than the objects from the BOSS database, and that they evoked comparable levels of interest. To do so, we conducted an online validation study, in which participants assessed their degree of familiarity of objects in each category, as well as their degree of interest in each object. As a supplementary question, we also asked participants to rate the degree to which they found each object appealing.

### Online validation study

#### Participants

435 participants completed this study, which was conducted on the Cloud Research platform^48^ (integrated with the Amazon Mechanical Turk; MTurk). We excluded 23 participants for attempting the study more than once. A further 22 were excluded for perseverative responses (e.g., providing the same response for every object). The final sample comprised 390 participants (205 self-identified males, 181 self-identified females, 2 non-binary, 2 unspecified), aged between 18 and 76 (mean = 40.32, SD = 12.12). All participants were from the United States, and were reimbursed US$2.20 for their time. Data were collected between August 2022 and January 2023. Participants provided informed consent, and study protocols were approved by Monash University’s Human Research Ethics Committee (ID: CF20/23934).

#### Experimental design

**Fig. 3:**
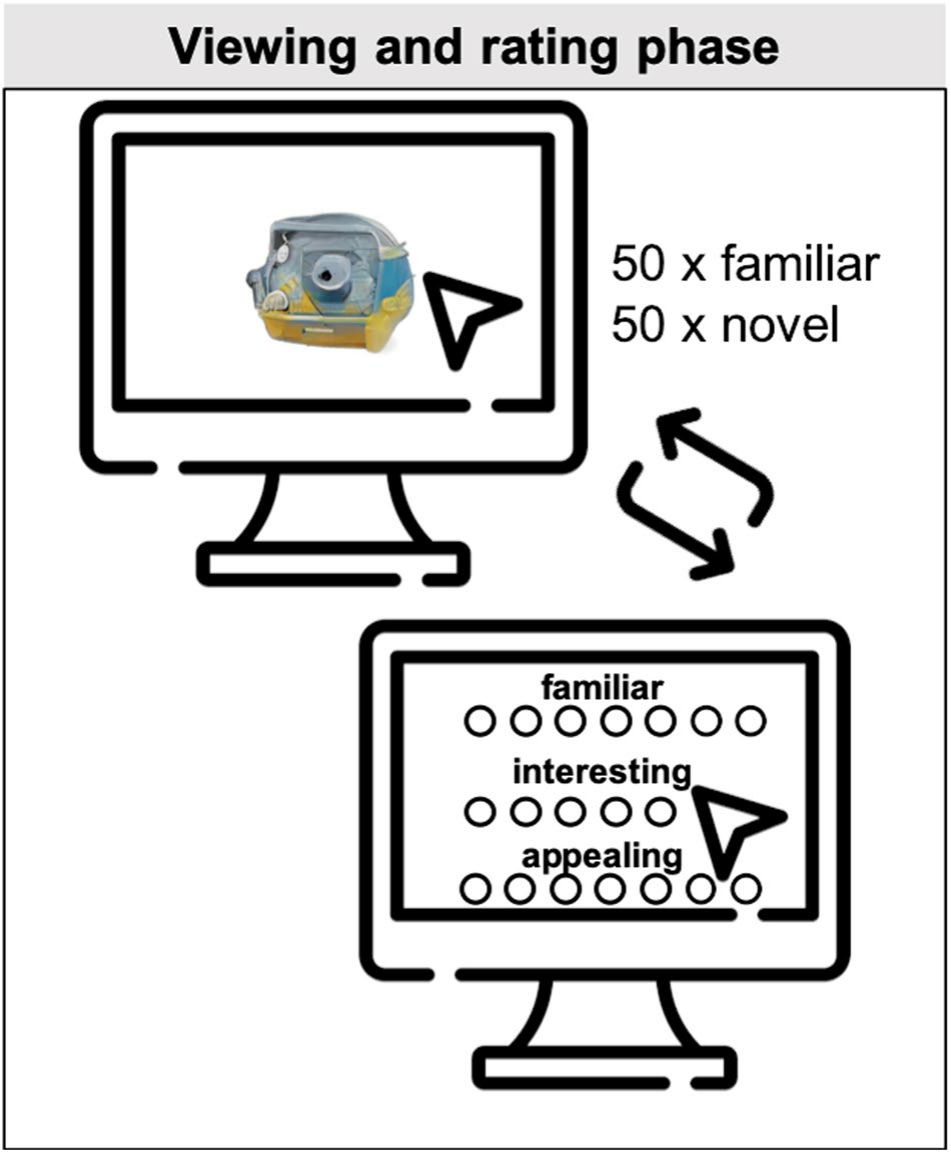
Overall study design. Participants viewed and rated a selection of 50 familiar and 50 novel items on familiarity, interest and appeal.

The task was implemented using jsPsych (version 7.2.1)^49^, with the experiment housed on https://cognition.run. To ensure that stimuli could be viewed with sufficient clarity and resolution, participants could only perform the task on a desktop or laptop computer (the task was aborted if an attempt was made to run it on a mobile phone). Each participant was required to rate a random subset of 50 familiar and 50 novel objects, which were presented in random order (Fig. 3).

Each trial was self-paced, and began with the object presented at the centre of the screen. Participants could freely view and interact with the object using their mouse. Objects could be stretched and rotated, and participants could pan and zoom into or out of each image. A reset button could be used to reset the image to its original configuration.

Once satisfied, participants pressed a “continue” button, and were presented with three questions: (1) “*How familiar is this object?*” (2) “*How interesting do you find this object?*” and (3) “*How appealing is this object?*” Responses were recorded on a 7-point Likert scale ranging from *Not at all* (1) to *Extremely* (7). Once all responses were complete, participants pressed a “continue” button, and the next object was then displayed.

#### Results

**Fig. 4:**
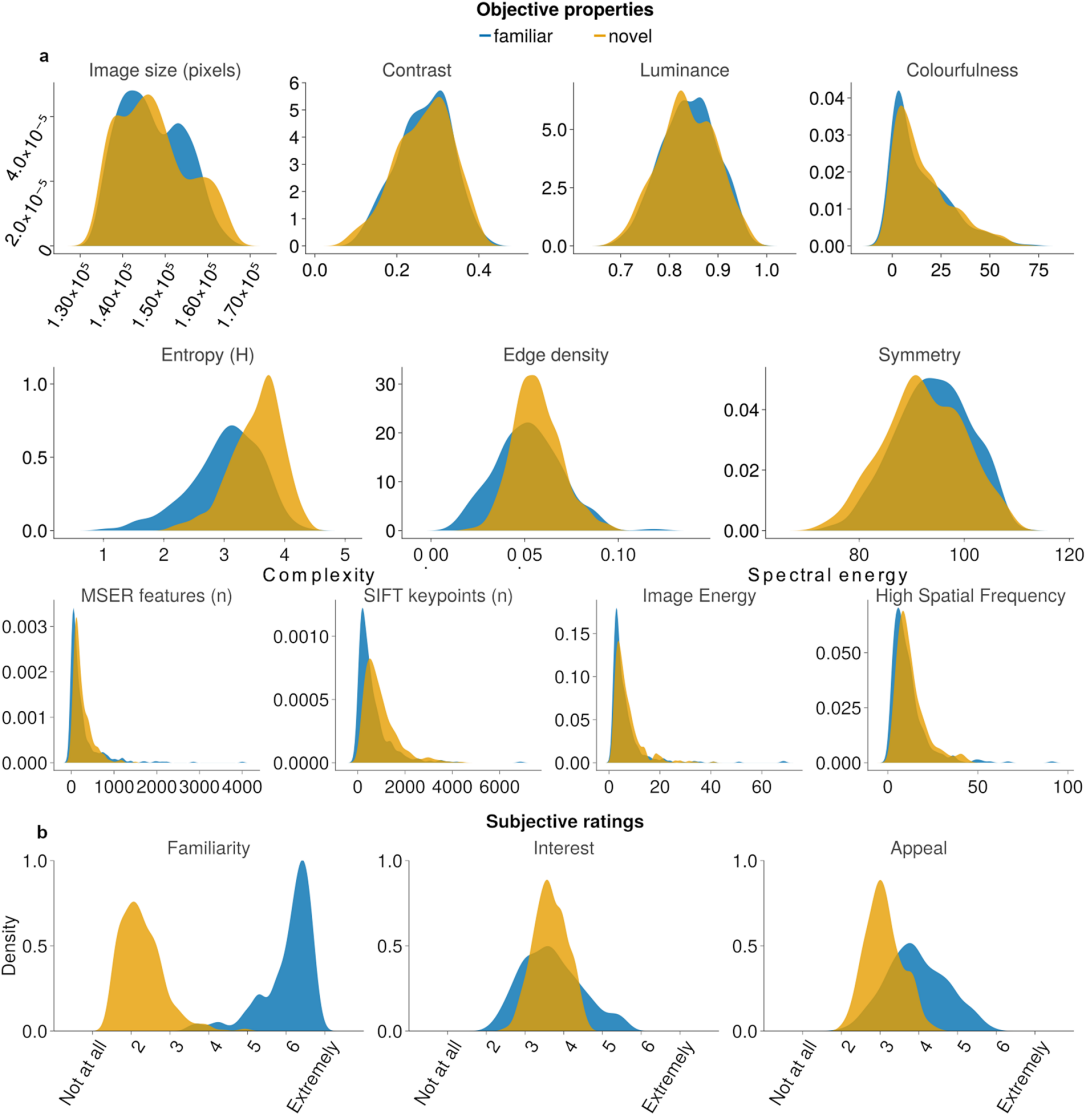
(**a**) Objective and (**b**) subjective properties of novel and familiar objects. Distributions visualised as density plots, with y-axes reflecting kernel density estimates. Note: MSER; maximally stable extremal regions, SIFT; scale-invariant feature transformation.

The key comparison was in familiarity ratings between novel and familiar objects. This provided decisive evidence that GAN-generated objects were indeed perceived as less familiar than the BOSS objects (Figs. 4b; novel_median_ = 2.19 ± 0.028 SEM, familiar_median_ = 6.26 ± 0.033; U = 134, p < 0.0001, BF_10_ = 5.10 × 10^21^). In addition, the novel and familiar objects evoked similar degrees of interest (novel = 3.63 ± 0.021, familiar = 3.61 ± 0.039, U = 78509, p = 0.788, BF_10_ = 0.085). Interestingly, we found familiar objects were more appealing than novel objects (novel = 3.04 ± 0.023, familiar = 3.87 ± 0.038, U = 28272, p < 0.0001, BF_10_ = 2.19 × 10^14^).

**Fig. 5:**
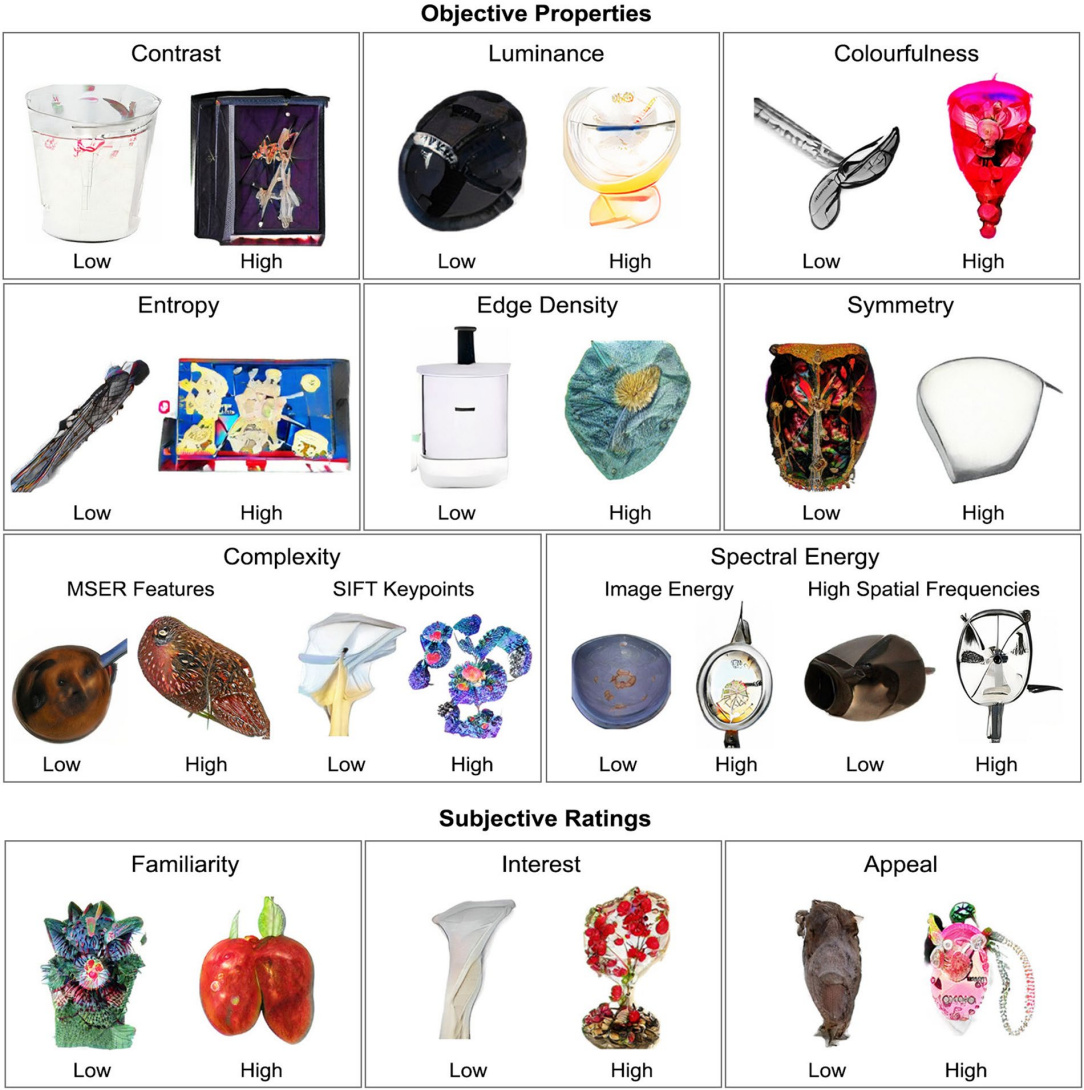
Exemplars of novel objects that are representative of those at the extreme of the distributions shown in Fig. 4.

In sum, the ‘IMAGINE’ dataset comprises 400 artificially-generated objects that are entirely plausible, yet do not in fact exist. These objects have been carefully standardised and matched to a complementary dataset of 400 familiar objects. Importantly, the novel objects are perceived by adult observers to be less familiar than the familiar objects, but similarly engaging. We hope that this stimulus set will facilitate future research in the neuroscience of object processing and perceptual novelty.

## Usage Notes

Users may choose to sample a subset of the familiar and novel stimuli for their unique purposes. Here, we have matched objects according to size, contrast, luminance and colourfulness. Although there may be significant statistical differences in symmetry and complexity across the corpus of novel and familiar objects as a whole, there is sufficient overlap between these features (Fig. 4) such that users will be able to sample a subset of novel and familiar objects which are matched across those features should they wish. Likewise, there may be sufficient variability within subjective ratings to use familiar and novel objects that are comparable on interest or appeal if required.

We also note that our online validation study comprised a group of participants that were exclusively based in the U.S.A. The subjective ratings of familiarity, interest and appeal are therefore limited to that population. Although we anticipate that most cultures would broadly consider the novel objects to be ‘less familiar’, the precise degree to which a novel stimulus is perceived as less familiar in other cultures, and the relative difference in ratings for novel and familiar objects, cannot be assumed.

Finally, we note that certain aspects of our procedure required manual inspection of the novel objects as a quality control measure. This was a critical aspect of our protocol to verify that the generated images were clear, plausible, distinct, and free of artefacts. Given recent advances in the development of artificial intelligence techniques, such steps could potentially be automated in the future.

## Data Availability

Software to generate novel objects is available at https://artbreeder.com. Code to perform data collection (i.e., run the online experiment) was created using jsPsych (version 7.2.1), and a modified version of the *mouse-tracking* extension (all available at^47^). Code to extract the objective properties of each object, and to compile the subjective ratings from our online study, was written in Python, MATLAB and Julia respectively (available at^47^).
